# An Analysis of HLA-A, -B, and -DRB1 Allele and Haplotype Frequencies of 21,918 Residents Living in Liaoning, China

**DOI:** 10.1371/journal.pone.0093082

**Published:** 2014-04-01

**Authors:** Xiao-Feng Li, Xu Zhang, Yang Chen, Kun-Lian Zhang, Xiang-Jun Liu, Jian-Ping Li

**Affiliations:** 1 HLA Laboratory, Liaoning Blood Center, Shenyang, Liaoning, China; 2 Liaoning Key Laboratory of Blood Safety Research, Shenyang, Liaoning, China; 3 BFR Diagnostics LTD, Beijing, China; 4 Shenyang Research Center for Stem Cell Clinical Application, Shenyang, Liaoning, China; Centers for Disease Control and Prevention, United States of America

## Abstract

HLA-A, -B and -DRB1 allele frequencies and their haplotype frequencies in 21,918 Chinese residents living in Liaoning Province, who were registered as volunteer donors of China Marrow Donor Registry, were investigated. They are composed of 93.37% Han Chinese, 5.1% Manchus, 0.57% Mongols, 0.46% Hui persons, 0.29% Koreans and 0.14% Xibe ethnic group. In total eighteen different HLA-A alleles, forty-eight different HLA-B alleles and fourteen different HLA-DRB1 alleles have been identified. Their frequencies are in agreement with the Hardy-Weinberg equilibrium. For Han Chinese in Liaoning, 1,534 different HLA-A-B-DRB1 haplotypes were identified, with a frequency of higher than 0.01%. A*30-B*13-DRB1*07, A*02-B*46-DRB1*09 and A*02-B*13-DRB1*12 are the most frequent haplotypes among Liaoning Han. While Liaoning Han, Liaoning Manchu, Liaoning Mongol, Liaoning Hui and Liaoning Korean share the northern Han characteristic haplotypes, all minority ethnic groups with the exception of Liaoning Manchu have developed their own unique HLA profiles. This dataset characterizes the HLA allele and haplotype frequencies in the Liaoning area and suggests that it is different from those in other parts of China and ethnic groups, which implicates transplant donor searching strategies and studies on population genetics.

## Introduction

Human Leukocyte Antigens (HLA) are located on the chromosome 6 in the 6P21.31 region and are the most polymorphic human genes with more than 8,000 alleles [1]. HLA genes play an important role in organ and Hematopoietic Stem Cell (HSC) transplantations. In order to provide stem cell transplant patients with more chances to find HLA-matched unrelated donors, China Marrow Donor Program (CMDP) launched the nationwide HLA typing program for marrow donor registry volunteers recruited over 23 different provinces in China in 2001. Our laboratory,contracted for HLA typing with CMDP, has typed about 22,000 volunteers from Liaoning Province for HLA-A, -B, -DRB1 loci from 2003 to 2006, and we uploaded the related data to the CMDP database annually. Up to now, CMDP has enlisted 1.8 million potential HSC donors and facilitated more than 3,800 HSC donations, including 127 donors from our Liaoning bank.

In earlier studies, we reported single locus HLA allele frequencies with a smaller sample size [2]–[4]. Here we present the largest HLA-A, -B and -DRB1 typing dataset in Liaoning Province. The samples were collected from 14 different cities throughout Liaoning Province. Although the entire samples were not randomly selected since they were exclusively potential donors for the China marrow donor bank, they contain a lot of useful information for analyzing the HLA gene diversity in Liaoning and its relationship with other parts of China.

Northern Han Chinese constitutes the main ethnic group of Liaoning, although many minority ethnic groups have lived here for thousand years. The Liaoning region was actually the home of over five millions of Manchu population who ruled China from 1616 to 1912 during the Qing Dynasty. There is also significant presence of Mongolian, Uyghur, Korean, Hui and Xibe ethnic populations in Liaoning. Our aim was to analyze the HLA allele and haplotype frequencies among Liaoning residents and compare them with other northern Chinese, southern Chinese, and minorities living outside Liaoning. The results are reported as below.

## Materials and Methods

### Ethics Statement

This project was approved by Medical Ethics Committee of Liaoning Blood Center, China. All participants provided their written informed consent and all clinical investigations were conducted according to the principles expressed in the Declaration of Helsinki.

### Characterization of Study Subjects

The volunteers were age 18–50 year old residents of the Liaoning, China who were randomly recruited through donor drives organized by local Red Cross societies in college campus, local community centers and shopping centers. The volunteers were multi-ethnic and consisted of Han Chinese, Manchu, Mongolians, Hui, Koreans, and Xibe men and women. The male/female ratio was 53.75%/46.25% in the study subjects.

### Samples and DNA Extraction

For each volunteer a sample of 5 ml whole blood was collected, divided equally, and stored at −80°C until processed. The DNA was extracted by using the mini column whole blood DNA extraction kit from Tiangen (Beijing, China). The DNA concentration was 40–80 ng/μl and optical density at 260/280 was 1.6–1.8.

### HLA Typing

All individuals were typed as HLA class I (HLA-A, and -B) and HLA class II (HLA-DRB1). For the Sequence Specific Primers (SSP) method, exons 1–4 of HLA-A and B loci and exon 2 of DRB1 locus were covered. For the Sequence Specific Oligonucleotide Probe (SSO) method, exons 2 & 3 of class I loci and exon 2 of class II locus were typed. Amplification was accomplished on the GeneAmp PCR system 9700 (Applied Biosystems, Foster City, CA, USA).

About a third of the samples were typed with the SSP method by means of the commercial kits from Life Technologies (Carlsberg, California, USA) and Biotest (Biotest AG, Dreieich, Germany). About two thirds of samples were typed with the SSO method by use of LABType HLA-A,-B,-DRB1 kits from One Lambda (Canoga Park, California, USA). The SSP typing results were analyzed with Unimatch plus 3.0 and Biotest HLA-SSP typing software provided by the manufactures, while the SSO results were analyzed by using HLATools software provided by One Lambda. When ambiguous results were produced from the SSP and SSO typing methods, the Sequencing Based Typing (SBT) kit -SBTexcellerator (Genome Diagnostics B.V., Utrecht, Netherlands) was applied to resolve unambiguous results. For HLA-A and -B, exons 2 to 4 were sequenced, and for DRB1 only exon 2 was sequenced. The HLA-SBT typing results were analyzed by means of SBTengine software 3.0 (Genome Diagnostics B.V., Utrecht, Netherlands). For the purpose of revolving ambiguous results at the serological level, the high-resolution group-specific SSP kits from Olerup SSP (Sweden) were deployed. Detail procedures were followed strictly according to the User Instructions provided by the kit manufacturers.

### Statistics Methods

Allele frequencies of HLA-A, -B and -DRB1 were calculated with the direct counting method. The deviation from Hardy-Weinberg equilibrium was calculated by χ^2^ test [5]. The haplotype frequencies were analyzed with the Arlequin software 3.5[6] based on the Expectation-Maximization (EM) algorithm [7]. The Linkage Disequilibrium (LD) of three-locus haplotype was calculated using the SPSS 13.0 software package according to the report [8], and the LD values, ranged from +1 to -1, reflect linkage disequilibrium intensity. The genetic distances between different populations were calculated as previously described by Nei [9] and a phylogenetic tree was constructed based on the allelic frequencies of HLA-A, -B and -DRB1 and the Neighbor-joining method with the PHYLIP-3.695 package.

### Consolidation of HLA Typing Results

The majority of DNA samples were typed at the intermediate resolution by the SSP and SSO methods. In order to consolidate the data analysis, all SSP, SSO and SBT data were converted into the first field resolution with the exception of B14, B15, B40 groups which were divided into corresponding serological groups.

## Results

### Ethnic Background of Volunteers

**Figure 1:**
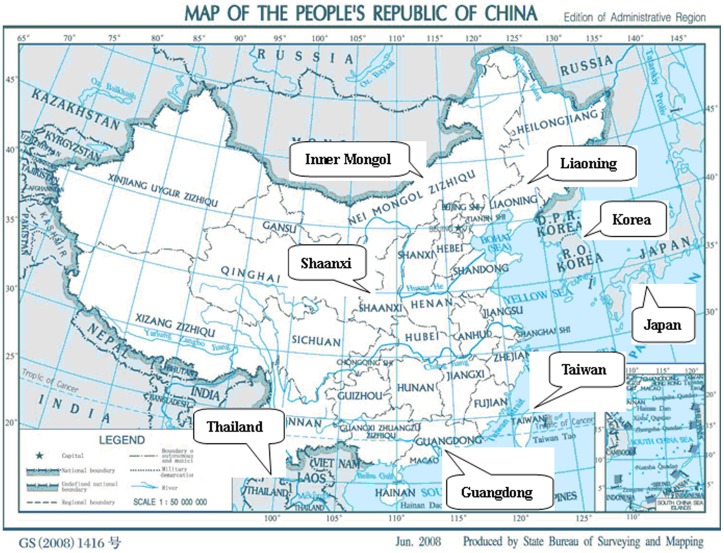
The Map of the People's Republic of China. The studied regions are marked with their name box.

Liaoning is a northeast province consisting of 84% Han Chinese and 16% minority Chinese. The location of Liaoning and its geographic neighbors are shown in Figure 1. The volunteers consisted of 93.37% Han Chinese, 5.1% Manchus, 0.57% Mongolians, 0.46% Hui persons, 0.29% Koreans and 0.14% Xibe ethnic persons (see Table 1). Overall Han Chinese is overrepresented by about 10%, while the minority groups areunderrepresented by about 50% relatively to their population size.

**Table 1:** Characterization of 21,918 bone marrow donor volunteers.

Ethnic Group	Sample N	Percentage %	Liaoning Population	Percentage %
Han	20465	93.37	36746911	84.00
Manchu	1118	5.10	5385000	12.31
Mongol	126	0.57	670000	1.53
Hui	101	0.46	264000	0.60
Korean	64	0.29	241052	0.55
Xibe	31	0.14	133000	0.30
Others	13	0.06	306360	0.70
**Total**	**21918**	**100**	**43746323**	**100**
Sex(Male/Female)	11778/10140	53.74/46.26		50.63/49.37

The ethnic information is based on the registration information filled by the volunteers. The Liaoning population information is based on the 2010 public survey results conducted and published by the Liaoning province government (http://www.gov.cn/gzdt/2011-05/13/content_1863235.htm).

### Frequencies of HLA-A, -B and - DRB1 Alleles in Liaoning Han Chinese

Among the 21,918 donors, 18 alleles for HLA-A, 48 alleles for HLA-B and 14 alleles for HLA-DRB1 locus were detected. Their frequencies are listed in Table 2. The distribution of the allele groups are in concordance with the Hardy-Weinberg equilibrium (p>0.05). In Liaoning Han Chinese, the 3 most frequent allele groups for each locus are respectively A*02, A*24, A*11, B*13, B*15(62), B*40(61), DRB1*15, DRB1*09 and DRB1*12. B15 is the most diverse HLA-antigen family in Liaoning Han Chinese. In total, 9 different serological groups within B15 family have been detected, includingB62(7.239%), B75(4.342%), B71(1.928%), B63(0.303%), B72(0.137%), B15(0.464%), B77(0.083%), B76(0.068%) and B70(0.032%). The following HLA alleles were found very rarely in Liaoning Han Chinese (<0.1%): A*25(0.042%), A*34(0.017%), A*66(0.090%), A*69(0.078%), A*74(0.015%, B*14(64)(0.081%), B15*(70)(0.032%), B*15(76)(0.068%), B*15(77)(0.083%), B*40(40)(0.015%),B*42((0.034%), B*47(0.034%), B*53(0.042%), B*73(0.027%) and B*78(0.002%). The following HLA alleles were not detected: A*36, A*43, A*80 and B*82.

**Table 2:** Gene frequencies (GF) of HLA-A, -B, and -DRB1 in 21,918 bone marrow donor volunteers in Liaoning by ethnic groups.

Alleles	Han % (N = 20465)	Manchu% (N = 1118)	Mongol% (N = 126)	Hui% (N = 101)	Korean% (N = 64)	Xibe% (N = 31)
A*01	4.686	5.501	6.746	1.980	3.906	3.226
A*02	32.883	33.274	31.349	33.663	34.375	27.419
A*03	4.630	3.801	5.159	5.941	1.563	3.226
A*11	15.935	15.206	13.095	10.891	14.063	19.355
A*23	0.330	0.134	0.397			1.613
A*24	16.015	17.039	15.079	17.327	19.531	8.065
A*25	0.042					
A*26	3.220	3.309	4.365	4.951	4.688	
A*29	0.904	0.581		0.990	0.781	1.613
A*30	7.166	7.021	5.556	6.931	4.688	14.516
A*31	4.173	4.875	7.143	5.941	4.688	4.839
A*32	1.573	1.521	1.984	2.475	0.781	4.839
A*33	7.286	6.574	7.143	7.921	10.156	8.065
A*34	0.017					
A*66	0.090	0.045				
A*68	0.955	0.984	1.587	0.990		3.226
A*69	0.078	0.134	0.397		0.781	
A*74	0.015					
Unknown	0.002					
B*07	3.494	2.996	2.778	1.980	3.906	3.226
B*08	0.909	0.626	0.794	0.495	0.781	1.613
B*13	12.167	12.567	12.698	12.871	6.250	17.742
B*14	0.002					
B*14(64)	0.081	0.045	0.794		0.781	
B*14(65)	0.330	0.268		2.475		
B*15	0.464	0.537		0.495		
B*15(62)	7.239	7.737	4.365	9.406	8.594	6.452
B*15(63)	0.303	0.179	0.794	0.990		1.613
B*15(70)	0.032	0.045				
B*15(71)	1.928	2.504	2.381	1.980	1.563	3.226
B*15(72)	0.137	0.134	0.397			
B*15(75)	4.342	4.025	1.984	4.951	3.125	6.452
B*15(76)	0.068	0.045				
B*15(77)	0.083	0.089				
B*18	0.540	0.447	0.794	2.475	0.781	
B*27	1.740	1.610	0.397	0.990	1.563	1.613
B*35	5.487	5.456	8.730	7.426	7.813	4.839
B*37	1.610	1.699	3.968	1.485	0.781	4.839
B*38	2.311	2.102	1.984	6.931	1.563	
B*39	1.906	1.878	2.778	2.475	3.125	
B*40	0.489	0.626	1.190		0.781	
B*40(40)	0.015	0.045				
B*40(60)	6.355	7.156	5.952	3.960	4.688	6.452
B*40(61)	7.515	7.290	8.730	4.951	9.375	3.226
B*41	0.173	0.045		0.990		
B*42	0.034	0.045				
B*44	5.089	4.070	4.762	2.475	4.688	6.452
B*45	0.132	0.045				
B*46	6.966	7.111	6.746	6.436	4.688	8.065
B*47	0.034					
B*48	3.819	3.444	1.984	2.970	5.469	4.839
B*49	0.188	0.089	0.794	0.495		
B*50	0.902	0.939	1.190	1.980	0.781	3.226
B*51	7.017	7.111	4.762	7.426	4.688	6.452
B*52	3.655	3.220	3.175	4.951	3.906	4.839
B*53	0.042	0.045				
B*54	3.132	3.846	2.778	0.990	4.688	
B*55	1.710	1.744	3.571		2.344	
B*55(54)	0.024					
B*56	0.279	0.268				
B*57	1.654	2.191	4.365	0.990	1.563	
B*58	4.298	4.562	2.778	3.465	6.250	4.839
B*59	0.098	0.089			3.125	
B*67	1.033	1.208	1.190	0.495	2.344	
B*73	0.027					
B*78	0.002					
B*81	0.147	0.045	0.397			
DRB1*01	2.71	1.97	4.37	6.44	2.34	4.84
DRB1*03	0.03	0.09	0.40			
DRB1*03(17)	3.59	4.03	2.78	4.46	3.13	4.84
DRB1*04	10.27	11.36	10.71	15.35	17.97	6.45
DRB1*07	11.66	12.48	13.49	11.88	7.81	25.81
DRB1*08	6.05	6.57	4.76	5.45	7.81	3.23
DRB1*09	13.56	13.28	11.90	10.40	11.72	11.29
DRB1*10	1.60	1.43	3.57	0.99	0.78	3.23
DRB1*11	6.64	5.50	6.35	6.44	7.03	8.06
DRB1*12	12.00	12.84	11.90	12.87	6.25	4.84
DRB1*13	6.31	5.23	4.76	3.96	7.81	4.84
DRB1*14	6.64	6.31	7.14	4.46	9.38	12.90
DRB1*15	17.37	17.31	15.87	15.84	17.97	9.68
DRB1*16	1.56	1.61	1.98	1.49		

### Frequencies of HLA-A, -B and - DRB1 Alleles of the Minority Ethnic Groups

The Manchu ethnic group constitutes the largest minority ethnic group in Liaoning with more than five million residents. Their HLA-A, -B, and -DRB1 alleles show high similarity to the Liaoning Han Chinese. The HLA allele frequencies vary less than 20% among alleles with a frequency greater than 0.5%. Currently, 670,000 Mongolians are living in Liaoning and make up 1.53% of total Liaoning population. In our study, 126 Mongolian samples were included. Their frequencies of A*01, A*68, B*37, B*57 and DRB1*10 are significantly higher than those in Liaoning Han, while those of B*15(62), B*15(75), B*27 and B*48 are much less common than those in Liaoning Han. For Hui ethnic group, their allele frequencies vary significantly from those of Liaoning Han. A*01, B*07, B*44, B*54, B*57 and B*67 are 50% to 300% less common in Liaoning Hui than in Liaoning Han, while their B*14(65), B*18, B*38, DRB1*01 are 70% to 250% more common in Liaoning Hui than in Liaoning Han. For Koreans living in Liaoning, A*03, A*32, B*13 and B*37 have significantly lower frequencies than those in Liaoning Han; on the contrary, the opposite is true for B*59, B*67 and DRB1*04. Only 31 samples with Xibe ethnic origin were included in our study subjects. Major variations in HLA allele frequencies between Xibe and the rest groups were observed, even so the frequency results of Xibe are not reliable due to their small sample size.

### Haplotype Frequencies of Liaoning Han Chinese

Based on the statistic analysis, 1,534 different HLA-A-B-DRB1 haplotypes were identified from 20,465 samples with frequencies of higher than 0.01%. Twenty-five of them are classified as frequent haplotypes based on a frequency of 0.5%. The most frequent haplotypes are A*30-B*13-DRB1*07(4.88%), A*02-B*46-DRB1*09 (2.38%) and A*02-B*13-DRB1*12 (1.61%). In Table 3, 50 most common HLA-A-B-DRB1 haplotypes are listed for Liaoning Han and other Liaoning ethnic groups. The distribution of Liaoning Han haplotypes based on their frequencies is shown in Table 4. The 7 most frequent haplotypes make up about 14% of total haplotype frequencies. It was found that 229 haplotypes had frequencies of higher than 0.1% and made up about 68% of the whole haplotype frequencies. The rare haplotypes with frequencies of lower than 0.01% contribute to 32% of total haplotype frequencies.

**Table 3:** HLA-A-B-DRB1 haplotypes of 21,918 bone marrow donor volunteers in Liaoning divided by ethnic groups.

Han	HF	Manchu	HF	Mongol	HF	Hui	HF	Korean	HF	Xibe	HF
A*30 B*13 DRB1*07	0.0488	A*30 B*13 DRB1*07	0.0503	A*30 B*13 DRB1*07	0.0476	A*30 B*13 DRB1*07	0.0446	A*02 B*15(62) DRB1*15	0.0391	A*30 B*13 DRB1*07	0.1290
A*02 B*46 DRB1*09	0.0238	A*02 B*46 DRB1*09	0.0193	A*02 B*13 DRB1*12	0.0388	A*02 B*13 DRB1*12	0.0396	A*33 B*58 DRB1*13	0.0391	A*02 B*51 DRB1*11	0.0484
A*02 B*13 DRB1*12	0.0161	A*33 B*58 DRB1*03(17)	0.0156	A*01 B*37 DRB1*10	0.0278	A*02 B*46 DRB1*09	0.0347	A*02 B*40(61) DRB1*09	0.0313	A*02 B*15(75) DRB1*09	0.0323
A*33 B*58 DRB1*03(17)	0.0131	A*02 B*13 DRB1*12	0.0140	A*02 B*46 DRB1*09	0.0278	A*24 B*15(62) DRB1*04	0.0345	A*02 B*46 DRB1*09	0.0313	A*02 B*15(75) DRB1*14	0.0323
A*33 B*58 DRB1*13	0.0116	A*33 B*58 DRB1*13	0.0130	A*24 B*40(61) DRB1*09	0.0235	A*33 B*14(65) DRB1*01	0.0248	A*30 B*13 DRB1*07	0.0313	A*02 B*40(60) DRB1*07	0.0323
A*02 B*46 DRB1*08	0.0115	A*01 B*57 DRB1*07	0.0122	A*01 B*57 DRB1*15	0.0159	A*02 B*15(75) DRB1*04	0.0198	A*33 B*44 DRB1*13	0.0313	A*02 B*48 DRB1*14	0.0323
A*33 B*44 DRB1*13	0.0114	A*02 B*46 DRB1*08	0.0118	A*02 B*35 DRB1*04	0.0159	A*11 B*52 DRB1*15	0.0198	A*33 B*58 DRB1*03(17)	0.0234	A*11 B*15(62) DRB1*04	0.0323
A*02 B*40(61) DRB1*09	0.0091	A*02 B*15(62) DRB1*15	0.0114	A*24 B*40(61) DRB1*15	0.0159	A*24 B*51(51) DRB1*09	0.0198	A*01 B*57 DRB1*07	0.0156	A*11 B*52 DRB1*15	0.0323
A*01 B*57 DRB1*07	0.0080	A*02 B*40(61) DRB1*12	0.0104	A*24 B*52 DRB1*15	0.0159	A*24 B*38 DRB1*15	0.0196	A*02 B*13 DRB1*12	0.0156	A*33 B*58 DRB1*03(17)	0.0323
A*01 B*37 DRB1*10	0.0079	A*11 B*40(60) DRB1*12	0.0101	A*24 B*40(60) DRB1*15	0.0152	A*02 B*15(71) DRB1*15	0.0149	A*02 B*35 DRB1*14	0.0156	A*01 B*15(71) DRB1*14	0.0161
A*02 B*15(75) DRB1*09	0.0079	A*11 B*15(62) DRB1*04	0.0096	A*02 B*40(60) DRB1*15	0.0125	A*02 B*51(51) DRB1*12	0.0149	A*02 B*40(61) DRB1*04	0.0156	A*01 B*37 DRB1*10	0.0161
A*02 B*15(62) DRB1*15	0.0078	A*02 B*40(61) DRB1*09	0.0095	A*01 B*15(62) DRB1*04	0.0119	A*24 B*13 DRB1*12	0.0149	A*02 B*40(61) DRB1*08	0.0156	A*02 B*13 DRB1*07	0.0161
A*11 B*15(62) DRB1*04	0.0073	A*02 B*35 DRB1*15	0.0092	A*02 B*35 DRB1*12	0.0119	A*33 B*58 DRB1*03(17)	0.0149	A*02 B*52 DRB1*15	0.0156	A*02 B*15(62) DRB1*09	0.0161
A*11 B*15(75) DRB1*12	0.0068	A*33 B*44 DRB1*13	0.0084	A*02 B*40(61) DRB1*08	0.0119	A*01 B*37 DRB1*10	0.0099	A*11 B*48 DRB1*11	0.0156	A*02 B*35 DRB1*04	0.0161
A*24 B*40(61) DRB1*09	0.0066	A*24 B*40(61) DRB1*09	0.0077	A*02 B*44 DRB1*07	0.0119	A*02 B*15(62) DRB1*04	0.0099	A*11 B*55 DRB1*04	0.0156	A*02 B*40(61) DRB1*11	0.0161
A*33 B*44 DRB1*07	0.0065	A*01 B*37 DRB1*10	0.0076	A*11 B*51(51) DRB1*09	0.0119	A*02 B*15(63) DRB1*13	0.0099	A*11 B*67 DRB1*15	0.0156	A*02 B*46 DRB1*08	0.0161
A*24 B*54 DRB1*04	0.0064	A*24 B*54 DRB1*04	0.0075	A*31 B*51(51) DRB1*16	0.0119	A*02 B*15(75) DRB1*09	0.0099	A*24 B*07 DRB1*01	0.0156	A*02 B*58 DRB1*13	0.0161
A*11 B*13 DRB1*12	0.0058	A*11 B*13 DRB1*12	0.0071	A*33 B*58 DRB1*03(17)	0.0119	A*02 B*15(75) DRB1*12	0.0099	A*24 B*35 DRB1*04	0.0156	A*03 B*07 DRB1*07	0.0161
A*02 B*40(61) DRB1*15	0.0055	A*11 B*46 DRB1*09	0.0070	A*33 B*58 DRB1*13	0.0119	A*02 B*18 DRB1*11	0.0099	A*24 B*40(60) DRB1*09	0.0156	A*03 B*44 DRB1*01	0.0161
A*03 B*07 DRB1*15	0.0055	A*02 B*50 DRB1*07	0.0069	A*24 B*13 DRB1*12	0.0089	A*02 B*35 DRB1*08	0.0099	A*24 B*46 DRB1*08	0.0156	A*11 B*07 DRB1*04	0.0161
A*11 B*52 DRB1*15	0.0053	A*02 B*15(75) DRB1*09	0.0068	A*02 B*40(61) DRB1*09	0.0082	A*02 B*38 DRB1*04	0.0099	A*24 B*48 DRB1*15	0.0156	A*11 B*13 DRB1*07	0.0161
A*02 B*40(60) DRB1*15	0.0052	A*24 B*40(61) DRB1*15	0.0067	A*02 B*07 DRB1*01	0.0079	A*02 B*39 DRB1*15	0.0099	A*24 B*52 DRB1*15	0.0156	A*11 B*15(71) DRB1*09	0.0161
A*24 B*40(61) DRB1*15	0.0051	A*02 B*15(71) DRB1*15	0.0065	A*02 B*13 DRB1*14	0.0079	A*02 B*46 DRB1*08	0.0099	A*24 B*59 DRB1*04	0.0156	A*11 B*35 DRB1*13	0.0161
A*02 B*40(61) DRB1*12	0.0051	A*02 B*51(51) DRB1*12	0.0064	A*02 B*15(62) DRB1*15	0.0079	A*02 B*51(51) DRB1*09	0.0099	A*01 B*07 DRB1*09	0.0078	A*11 B*40(60) DRB1*15	0.0161
A*02 B*51(51) DRB1*09	0.0051	A*24 B*13 DRB1*12	0.0064	A*02 B*15(71) DRB1*15	0.0079	A*02 B*54 DRB1*04	0.0099	A*01 B*40(60) DRB1*11	0.0078	A*11 B*46 DRB1*09	0.0161
A*02 B*15(75) DRB1*15	0.0049	A*11 B*13 DRB1*15	0.0063	A*02 B*39 DRB1*04	0.0079	A*03 B*35 DRB1*03(17)	0.0099	A*01 B*52 DRB1*15	0.0078	A*11 B*46 DRB1*12	0.0161
A*24 B*15(62) DRB1*04	0.0048	A*02 B*40(61) DRB1*15	0.0062	A*02 B*40(61) DRB1*14	0.0079	A*03 B*35 DRB1*07	0.0099	A*02 B*07 DRB1*01	0.0078	A*11 B*48 DRB1*14	0.0161
A*11 B*13 DRB1*15	0.0046	A*11 B*15(75) DRB1*12	0.0062	A*02 B*46 DRB1*11	0.0079	A*03 B*51(51) DRB1*04	0.0099	A*02 B*13 DRB1*08	0.0078	A*23 B*37 DRB1*14	0.0161
A*32 B*52 DRB1*15	0.0046	A*02 B*51(51) DRB1*04	0.0061	A*03 B*35 DRB1*07	0.0079	A*11 B*15(62) DRB1*04	0.0099	A*02 B*15(62) DRB1*04	0.0078	A*24 B*15(62) DRB1*12	0.0161
A*02 B*15(75) DRB1*12	0.0046	A*02 B*40(60) DRB1*15	0.0054	A*03 B*44 DRB1*13	0.0079	A*24 B*35 DRB1*12	0.0099	A*02 B*15(75) DRB1*09	0.0078	A*24 B*40(60) DRB1*08	0.0161
A*02 B*48 DRB1*09	0.0044	A*11 B*51(51) DRB1*09	0.0053	A*03 B*46 DRB1*12	0.0079	A*26 B*38 DRB1*01	0.0099	A*02 B*15(75) DRB1*14	0.0078	A*24 B*40(61) DRB1*15	0.0161
A*03 B*44 DRB1*13	0.0043	A*24 B*13 DRB1*07	0.0052	A*11 B*07 DRB1*15	0.0079	A*31 B*15(62) DRB1*15	0.0099	A*02 B*27 DRB1*12	0.0078	A*24 B*46 DRB1*09	0.0161
A*24 B*13 DRB1*12	0.0042	A*02 B*67 DRB1*12	0.0051	A*11 B*15(62) DRB1*08	0.0079	A*31 B*35 DRB1*09	0.0099	A*02 B*35 DRB1*04	0.0078	A*24 B*50 DRB1*12	0.0161
A*24 B*35 DRB1*15	0.0041	A*02 B*40(60) DRB1*09	0.0051	A*11 B*35 DRB1*01	0.0079	A*32 B*40(61) DRB1*15	0.0099	A*02 B*35 DRB1*11	0.0078	A*29 B*35 DRB1*11	0.0161
A*02 B*35 DRB1*15	0.0041	A*02 B*48 DRB1*15	0.0050	A*11 B*35 DRB1*08	0.0079	A*33 B*13 DRB1*07	0.0099	A*02 B*35 DRB1*15	0.0078	A*30 B*44 DRB1*13	0.0161
A*11 B*40(60) DRB1*12	0.0040	A*02 B*15(62) DRB1*04	0.0049	A*11 B*35 DRB1*14	0.0079	A*24 B*15(62) DRB1*15	0.0051	A*02 B*38 DRB1*09	0.0078	A*31 B*27 DRB1*01	0.0161
A*24 B*15(62) DRB1*15	0.0040	A*02 B*54 DRB1*04	0.0049	A*11 B*40(60) DRB1*12	0.0079	A*24 B*38 DRB1*04	0.0051	A*02 B*38 DRB1*15	0.0078	A*31 B*46 DRB1*09	0.0161
A*02 B*15(62) DRB1*04	0.0040	A*03 B*07 DRB1*15	0.0049	A*11 B*55(55) DRB1*04	0.0079	A*01 B*41 DRB1*15	0.0050	A*02 B*39 DRB1*08	0.0078	A*31 B*51 DRB1*15	0.0161
A*02 B*15(62) DRB1*12	0.0039	A*02 B*46 DRB1*04	0.0045	A*24 B*13 DRB1*07	0.0079	A*01 B*57 DRB1*07	0.0050	A*02 B*40 DRB1*07	0.0078	A*32 B*15(63) DRB1*07	0.0161
A*02 B*48 DRB1*15	0.0039	A*33 B*44 DRB1*07	0.0044	A*24 B*57 DRB1*07	0.0079	A*02 B*07 DRB1*07	0.0050	A*02 B*48 DRB1*04	0.0078	A*32 B*44 DRB1*07	0.0161
A*02 B*13 DRB1*07	0.0038	A*24 B*15(62) DRB1*04	0.0044	A*31 B*15(71) DRB1*04	0.0079	A*02 B*07 DRB1*08	0.0050	A*02 B*48 DRB1*11	0.0078	A*32 B*44 DRB1*14	0.0161
A*11 B*40(60) DRB1*09	0.0038	A*02 B*48 DRB1*12	0.0044	A*33 B*15(63) DRB1*07	0.0079	A*02 B*13 DRB1*14	0.0050	A*02 B*51 DRB1*04	0.0078	A*33 B*13 DRB1*15	0.0161
A*02 B*51(51) DRB1*14	0.0037	A*24 B*15(62) DRB1*12	0.0044	A*33 B*44 DRB1*07	0.0079	A*02 B*13 DRB1*15	0.0050	A*02 B*51 DRB1*14	0.0078	A*33 B*50 DRB1*07	0.0161
A*11 B*51(51) DRB1*09	0.0036	A*02 B*40(60) DRB1*11	0.0043	A*33 B*44 DRB1*15	0.0079	A*02 B*15(62) DRB1*11	0.0050	A*02 B*54 DRB1*04	0.0078	A*33 B*52 DRB1*01	0.0161
A*02 B*15(71) DRB1*04	0.0036	A*24 B*51(51) DRB1*09	0.0042	A*01 B*13 DRB1*07	0.0040	A*02 B*15(62) DRB1*15	0.0050	A*02 B*55 DRB1*04	0.0078	A*68 B*08 DRB1*03(17)	0.0161
A*24 B*46 DRB1*09	0.0035	A*24 B*40(60) DRB1*15	0.0042	A*01 B*49 DRB1*04	0.0040	A*02 B*27 DRB1*08	0.0050	A*02 B*59 DRB1*09	0.0078	A*68 B*37 DRB1*10	0.0161
A*11 B*40(60) DRB1*15	0.0035	A*30 B*13 DRB1*04	0.0042	A*01 B*57 DRB1*13	0.0040	A*02 B*38 DRB1*12	0.0050	A*02 B*67 DRB1*14	0.0078		
A*24 B*40(61) DRB1*12	0.0034	A*02 B*51(51) DRB1*15	0.0042	A*02 B*08 DRB1*03(17)	0.0040	A*02 B*40(61) DRB1*03(17)	0.0050	A*03 B*15(62) DRB1*04	0.0078		
A*02 B*50 DRB1*07	0.0034	A*02 B*15(75) DRB1*12	0.0041	A*02 B*08 DRB1*12	0.0040	A*02 B*44 DRB1*13	0.0050	A*03 B*27 DRB1*04	0.0078		
A*02 B*46 DRB1*12	0.0034	A*02 B*46 DRB1*11	0.0041	A*02 B*15(71) DRB1*09	0.0040	A*02 B*46 DRB1*01	0.0050	A*11 B*15(62) DRB1*12	0.0078		

Only top 50 frequent haplotypes of each ethnic group are displayed. HF: Haplotypes Frequency.

**Table 4:** Distribution of HLA-A-B-DRB1 haplotypes in Liaoning Han (N = 20,465).

Frequency Range	Number of Unique Haplotypes	% of Total Haplotypes
>0.01	7	13.62
0.01–0.001	222	54.20
0.001–0.0001	1305	29.65
Total	1534	97.47

Linkage disequilibrium (LD) is one of the most important characteristics of the HLA system. Forty-nine of the 50 commonest haplotypes in the Liaoning Han population were in positive LD (see Table 5). A*30-B*13-DRB1*07, A*02-B*46-DRB1*09, A*02-B*13-DRB1*12, A*33-B*58-DRB1*03(17), A*33-B*58-DRB1*13, A*02-B*46-DRB1*08, and A*33-B*44-DRB1*13 are common haplotypes with the significant linkage disequilibrium (both haplotype frequency and LD are higher than 1%).

**Table 5:** Linkage disequilibrium (LD) of HLA-A-B-DRB1 haplotypes in Liaoning Han (N = 20,465).

	Haplotype	HF	LD
1	A*30 B*13 DRB1*07	4.88%	4.7783%
2	A*02 B*46 DRB1*09	2.38%	2.0694%
3	A*02 B*13 DRB1*12	1.61%	1.1299%
4	A*33 B*58 DRB1*03(17)	1.31%	1.2988%
5	A*33 B*58 DRB1*13	1.16%	1.1402%
6	A*02 B*46 DRB1*08	1.15%	1.0114%
7	A*33 B*44 DRB1*13	1.14%	1.1166%
8	A*02 B*40(61) DRB1*09	0.91%	0.5749%
9	A*01 B*57 DRB1*07	0.80%	0.7910%
10	A*01 B*37 DRB1*10	0.79%	0.7888%
11	A*02 B*15(75) DRB1*09	0.79%	0.5964%
12	A*02 B*15(62) DRB1*15	0.78%	0.3665%
13	A*11 B*15(62) DRB1*04	0.73%	0.6115%
14	A*11 B*15(75) DRB1*12	0.68%	0.5970%
15	A*24 B*40(61) DRB1*09	0.66%	0.4968%
16	A*33 B*44 DRB1*07	0.65%	0.6068%
17	A*24 B*54 DRB1*04	0.64%	0.5885%
18	A*11 B*13 DRB1*12	0.58%	0.3473%
19	A*02 B*40(61) DRB1*15	0.55%	0.1208%
20	A*03 B*07 DRB1*15	0.55%	0.5219%
21	A*11 B*52 DRB1*15	0.53%	0.4288%
22	A*02 B*40(60) DRB1*15	0.52%	0.1570%
23	A*24 B*40(61) DRB1*15	0.51%	0.3009%
24	A*02 B*40(61) DRB1*12	0.51%	0.2135%
25	A*02 B*51 DRB1*09	0.51%	0.1971%
26	A*02 B*15(75) DRB1*15	0.49%	0.2420%
27	A*24 B*15(62) DRB1*04	0.48%	0.3609%
28	A*11 B*13 DRB1*15	0.46%	0.3069%
29	A*32 B*52 DRB1*15	0.46%	0.4598%
30	A*02 B*15(75) DRB1*12	0.46%	0.2887%
31	A*02 B*48 DRB1*09	0.44%	0.2443%
32	A*03 B*44 DRB1*13	0.43%	0.4151%
33	A*24 B*13 DRB1*12	0.42%	0.1862%
34	A*24 B*35 DRB1*15	0.41%	0.2574%
35	A*02 B*35 DRB1*15	0.41%	0.0966%
36	A*11 B*40(60) DRB1*12	0.40%	0.2785%
37	A*24 B*15(62) DRB1*15	0.40%	0.1986%
38	A*02 B*15(62) DRB1*04	0.40%	0.1555%
39	A*02 B*15(62) DRB1*12	0.39%	0.1044%
40	A*02 B*48 DRB1*15	0.39%	0.1719%
41	A*02 B*13 DRB1*07	0.38%	−0.0865%
42	A*11 B*40(60) DRB1*09	0.38%	0.2427%
43	A*02 B*51 DRB1*14	0.37%	0.2168%
44	A*11 B*51 DRB1*09	0.36%	0.2084%
45	A*02 B*15(71) DRB1*04	0.36%	0.2949%
46	A*24 B*46 DRB1*09	0.35%	0.1987%
47	A*11 B*40(60) DRB1*15	0.35%	0.1741%
48	A*24 B*40(61) DRB1*12	0.34%	0.1956%
49	A*02 B*50 DRB1*07	0.34%	0.3054%
50	A*02 B*46 DRB1*12	0.34%	0.0651%

### Haplotypes of Different Ethnic Groups

Focusing on the ten most frequent haplotypes of Liaoning Han (marked in bold in Table 3), the Liaoning Manchus share seven of them; the Liaoning Mongols share five of them; the Liaoning Hui persons share three of them; the Liaoning Koreans share eight and Liaoning Xibe persons share only two of them. Each ethnic group shows their unique common haplotypes. For Liaoning Koreans, A*02-B*15(62)-DRB1*15 is the most common haplotype, but it is not shared by any other ethnic groups within the top seven haplotypes. Haplotypes specific for Liaoning Mongols include A*24-B*40(61)-DRB1*09, A*02-B*35-DRB1*04, A*24-B*40(61)-DRB1*15 and A*24-B*52-DRB1*15. Haplotypes only common for Liaoning Hui are A*24-B*15(62)-DRB1*04, A*33-B*14(65)-DRB1*01, A*02-B*15(75)-DRB1*04 and A*11-B*52-DRB1*15. The Xibe ethnic group is exceptional because they have several common haplotypes not found in the commonest 50 haplotypes of any other Liaoning ethnic group. A few examples are A*02-B*51-DRB1*11, A*02 -B*40(60)-DRB1*07 and A*02 B*48- DRB1*14.

### Genetic Distance and Phylogenetic Tree

**Figure 2:**
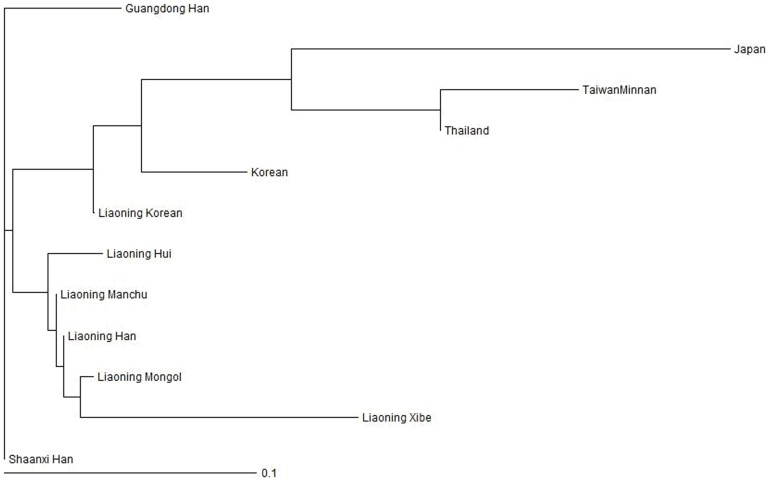
Phylogenetic Tree constructed by the neighbor-joining method display the relationship between Liaoning Han, other Liaoning ethnic groups included in our study, Guangdong Han, Shaanxi Han and populations from Korea, Thailand, Taiwan (Minnan) and Japan.

The phylogenetic tree is constructed with HLA-A, -B and -DRB1 allele frequencies presented in Table 2 and the published data for the northern Han Chinese from the Shaanxi region [10], southern Han Chinese from the Guangdong region [11], Koreans from Seoul Korea [12], Minnan ethnic group from Taiwan [13], Thai people in Thailand [14] and Japanese in Japan [15]. Liaoning is a province in the northeastern part of China as shown in the map of China (Figure 1). Shaanxi is located in northwestern China, which was the center of ancient Chinese history. Guangdong Province resides at the very southern end of mainland China. Korea is adjacent to Liaoning on its southeast side while Mongolia is adjacent to Liaoning on its northwest side. Taiwan, Thailand and Japan represent other parts of Southeast Asia. Genetic distance here measures genetic divergence between populations within a species. A smaller genetic distance indicates a closer genetic relationship between two populations, whereas a larger genetic distance indicates a further genetic relationship [16]. The study on genetic distances between Liaoning Han and the rest of study groups revealed that Liaoning Manchus have the shortest genetic distance to Liaoning Han Chinese (0.002084), followed by Liaoning Mongols (0.015929), Shaanxi Han Chinese (0.020565), Liaoning Hui persons (0.027006), Liaoning Koreans (0.036637), Guangdong Han Chinese (0.065632), Liaoning Xibe persons (0.107017), Seoul Koreans (0.108577), Thai (0.151685), Taiwan Minnan (0.228235) and Japanese (0.260845). The phylogenetic tree is shown in Figure 2.

## Discussion

The technology of HLA typing has significantly evolved since the PCR method was introduced by Mullis and Faloona [17]. Nowadays, there are several kinds of PCR-based HLA typing being used: DNA amplification with sequence specific primers (SSP) [18], sequence specific oligonucleotide probes (SSO) [19], single-stranded conformation polymorphism (SSCP) [20], sequence-based typing (SBT) [21], and DNA chip technology [22]. These HLA typing techniques are of much higher accuracy as well as reliability than the conventional serologic typing method, and also may facilitate the standardization of HLA typing processes [23]. The SSP and SSO methods are low-intermediate resolution techniques at two-digit level. The advantage of the SSO method based on Luminex system lies in its high throughput, because 96 samples can be completed in one day by a skilled technician. SBT is a gold-standard [24], high-resolution method at the four-digit level that was used to resolve theambiguous types and to identify novel alleles. In this paper, the data of HLA genotyping from 2003 to 2006 were based on the SSP and SSO methods. The SBT method was used only to resolve the ambiguous types and to identify novel alleles. In 2007, we began to use the SBT method in the routine HLA genotyping. The high-resolution data are being analyzed and will be reported in another paper.

HLA functions as a useful tool to clarify the differences between genetic makeup of Liaoning residents and other parts of China. The Han Chinese is divided into two major groups, namely northern Han and southern Han [25]. The HLA allele frequencies of the Liaoning Han show close similarity to the frequencies of the Shaanxi Northern Han, because both have less than 20% variation in allele frequencies. Shaanxi played a central role in ancient northern Chinese history for thousands of years. This is predictable given that Liaoning is one of the three northeastern Chinese provinces that was populated by the geographic expansion of the Northern Han Chinese.

There are striking similarities between Liaoning Han and Liaoning Manchu ethnic group in HLA allele and haplotype frequencies. This genetic closeness is also supported by the genetic distance calculation. This can be explained by the fact that Manchus are descended from the Jurchen people with North East Asia origin (http://en.wikipedia.org/wiki/Manchu_people#Origins_and_early_history). There is difference in Manchu HLA allele frequencies between our study and previous one [26]. For example, A*33, B*38, B*46, and DRB*14 have frequencies of 6.57%, 2.10%, 7.11%, 6.31% in the current study, but 2.8%, 0.9%, 4.1% and 12.3% in the previous study conducted with Manchu residents living in Harbin. Harbin is located about 600 km to the north of Liaoning. It is not clear about what contributes to this difference beside different geographic locations. Liaoning was the historic home for Manchu. It is possible that more intensive gene exchange took place between Liaoning Han and Liaoning Manchu in the Liaoning region. The HLA allele frequencies of Liaoning Mongol in our dataset are similar to those published previously [27], [28]. Both B*37 and B*57 distinguish Mongol from the northern Han based on much higher frequencies in Mongol.

The HLA haplotype analysis is widely used in human population genetics, anthropological studies, as well as the optimal marrow donor bank size planning. It carries more specific information than allele frequencies. A number of studies have addressed the Chinese HLA allele and haplotype distribution from different regions including Sichuan, Jiangsu, Fujian, Guangdong, Xi'an, Yunan [29]–[34]. However, there is no report of any large sample study about HLA haplotypes in Liaoning.

Linkage disequilibrium is the common characteristic of HLA genetics. This is true for Liaoning Han population according to Table 5. The top four most common haplotypes found among Liaoning Han in this study include A*30-B*13-DRB1*07, A*02-B*46-DRB1*09, A*02-B*13-DRB1*12 and A*33-B*58-DRB1*03(17) which actually consist of allele groups ranking the 5^th^ (A*30, B*46) and 10^th^ (B*58, DRB1*03(17)) positions in the allele frequency ranking (Table 2). B*57 and B*37 rank at the 20^th^ and 21^st^ places in the frequency list; but their corresponding haplotypes, A*01-B*37-DRB1*10 and A*01-B*57-DRB1*07 ranked within the top 10 most frequent haplotypes. The LDs are similar to the findings in the studies conducted in Shaanxi and Inner Mongolia, China [10], [28].

The fact that HLA-A-B-DRB1 haplotype frequencies of Liaoning Han show high similarities to those in Shaanxi can be expected since both of them belong to the northern China regions (the North of Yangtze River). There is below 30% difference in haplotype frequencies among 18 out of the top 20 haplotypes when Liaoning is compared with Shaanxi (The data were not shown).

Korea is adjacent to Liaoning on its southeast side. Our haplotype data do show that the Korean population is much closer to the northern Han than to the southern Han. Nine out of top 10 haplotype rankings in Liaoning Han are also listed in the top 10 most frequent haplotypes in Liaoning Korean. Korean do have own unique haplotypes that show a very different frequency pattern compared to Han. The most common haplotype A*02-B*15(62)-DRB1*15 in Liaoning Korean is five-time less frequent than that in Liaoning Han (Table 3). A*33-B*58-DRB1*13 (3.91%) and A*33-B*44-DRB1*13 (3.13%), the very common haplotypes in Liaoning Korean, reflect also a 3-fold lower frequency in Liaoning Han.

It is well known that A*30-B*13-DRB1*07 is the most common haplotype in the northern Han Chinese, while A*02-B*46-DRB1*09 is the most common haplotype in the southern Han Chinese [29]–[34]. Indeed, the A*30-B*13-DRB1*07 turned out to be the most common haplotype in all ethnic groups in our study with the exception of Liaoning Korean.

A*02-B*35-DRB1*04 and A*24-B*52-DRB1*15, two high-frequency haplotypes appear to be unique to the Mongolian population, i.e. both of them clearly present a lower frequency in Liaoning and Shaanxi Han, but much less in southern Han (The data were not shown).

Our phylogenetic tree study has placed Liaoning Han, Liaoning Manchu, Liaoning Mongol, Liaoning Hui and Liaoning Xibe in the same close cluster as expected. In addition to the Liaoning Han cluster, Liaoning Hui differs substantially from others. A*01 is significantly less common in Liaoning Hui than in Liaoning Han (1.980% vs. 4.686%), and B*14(65), B*18 and B*38 are 3–7 times more common in Liaoning Hui than in Liaoning Han volunteers. We have noted that Liaoning Manchu, Liaoning Hui, Liaoning Korean are quite different in their HLA profiles from the same ethnic groups reported previously. The differences between Liaoning Manchu and Harbin Manchu were already discussed. Liaoning Hui also differs in many alleles from Qinghai Hui [26]. A*30, A*31, B*14(65), and B*18 show 2.5–12-fold higher allele frequencies in Liaoning Hui than in Qinghai Hui, while A*01, B*27, and B*40(61) behave in the opposite way. Liaoning Korean display a large genetic distance to Liaoning Han, but it clearly differs from the Korean residents in Seoul, Korea [11]. It is possible that Liaoning Koreans have undergone substantial gene exchange with the Liaoning Han population in the Liaoning region and have acquired certain HLA characteristics from the Liaoning Han while still maintaining Korean unique HLA gene composition. The genetic distance between Shaanxi Han and Liaoning Han is three times shorter than that between Guangdong Han and Liaoning Han. This explains the fact that both Shaanxi and Liaoning belong to northern China while Guangdong represents a southern China province. Thailand, Taiwan/Minnan and Japan lie relative close to each other in the same branch since they are all from the Southeast Asia region. Japan population shows the most distinct features with the longest distance from the rest of all the study groups. This can be explained with the fact that Japan is most isolated nation from their neighbors during the thousand years of history.

Finally it is important to emphasize that our data may not reflect the true ethnic makeup of the Liaoning population due to the fact that the minority ethnic groups are less represented in the marrow donor registry population. The frequency data are also based on local bone marrow donor volunteers who could be a biased group in terms of representing the entire Liaoning population. We have no way to verify the ethnicity of samples from minority groups.

In summary, we have analyzed HLA-A-B-DRB1 allele and haplotype frequencies of Liaoning residents based on the local marrow donor registry volunteers classified by various ethnic backgrounds and compared to the populations from other parts of China including its geographic neighbors, Koreans and Mongolians. The HLA haplotypes of Liaoning Han carry a clear Northern Han signature while other ethnic groups possess their unique characteristics except Liaoning Manchu who show almost identical HLA-A, -B and -DRB1 allele frequencies to those of Liaoning Han. Liaoning Mongolians and Koreans show clearly more similarities to Liaoning Han than to southern Han in respect to their HLA haplotypes. No specific haplotype has been found to be uniquely shared between Liaoning and Koreans or between Liaoning and Mongolians.
